# Lupoid cutaneous leishmaniasis in Afghanistan treated with 0.045% DAC N-055

**DOI:** 10.1186/1753-6561-5-S6-P247

**Published:** 2011-06-29

**Authors:** KW Stahl, IG Sakhayeee

**Affiliations:** 1 Waisenmedizin e. V., Freiburg i. Brsg., Germany; 2 Dermatology, Balkh Civil Hospital, Mazar-e-Sharif, Afghanistan

## Introduction / objectives

Lupoid leishmaniasis (LCL) is a rare, face disfiguring, treatment resistant complication which has responded to the expensive photodynamic therapy in 5 patients in Iran [ F. Ghaffarifar et al. La Rev. de la Méditerranée orientale Vol. 12 No. 6, 2006]. Here we report 5 cases,- photodocumented as best as we could under the actually disastrous conditions of Afghan Health Care,- treated with 0.045% DAC N-055 [German Drug Codex, 1990], which is split into ^1^O_2_ and 2NaClO_2_ after 360 nm light exposure and reacts as chlorite in iodometry.

## Methods

The faces of 5 patients with a CL anamnesis and clinically diagnosed as lupoïd CL were treated daily for the first week with 0.045 % DAC N-055 prepared in basic crème DAC B-20 under occlusive dressings, thereafter the patients were instructed to treat themselves with this hydrophilic crème and were told to show up as outdoor patients at least once per fortnight. Pharmaceutical chlorite, NaClO_2_ [monograph DAC N-055] is O_2_ enriched due to its Na_2_Cl_2_O_6_ content, formerly called TCDO, (NaClO_2_)_4_·O_2_, which fights viral, bacterial or parasitic tissue infections and promotes wound granulation even in radiogenic ulcers [http://www-pub.iaea.org/mtcd/publications/pdf/te_1300_web.pdf].

## Results

1 patient was lost, 4 patients healed completely without scarring and with excellent cosmetic results (see photos!), 3 within 3 months, 1 after 10 months because of an additional severe eczema. There were no clinical signs of recurrence, one patient was followed for 6 years.

## Conclusion

The low treatment costs for such excellent cosmetic results are appropriate for poor Afghan patients.

## Disclosure of interest

None declared.

